# Effects of enamel matrix derivative in nonsurgical periodontal therapy on pro-inflammatory profiles, microbial environment and clinical outcome: a randomized clinical trial

**DOI:** 10.1007/s00784-023-05254-1

**Published:** 2023-10-16

**Authors:** Christian Wehner, Dino Tur, Gerlinde Durstberger, Markus Laky, Brenda Laky, Oleh Andrukhov, Andreas Moritz, Xiaohui Rausch-Fan

**Affiliations:** 1 Division of Conservative Dentistry and Periodontology, University Clinic of Dentistry, Medical University of Vienna, Sensengasse 2A, 1090 Vienna, Austria; 2 Austrian Research Group for Regenerative and Orthopedic Medicine (AURROM), Vienna, Austria; 3 MedSciCare, Vienna, Austria; 4 Competence Center for Periodontal Research, University Clinic of Dentistry, Medical University of Vienna, Sensengasse 2A, 1090 Vienna, Austria; 5 Division for Dental Student Training and Patient Care, University Clinic of Dentistry, Medical University of Vienna, Sensengasse 2A, 1090 Vienna, Austria; 6 Clinical Research Center, University Clinic of Dentistry, Medical University of Vienna, Sensengasse 2A, 1090 Vienna, Austria

**Keywords:** Periodontitis, Nonsurgical therapy, Enamel matrix derivative, Gingival crevicular fluid, Inflammation

## Abstract

**Objectives:**

This study aimed to evaluate the impact of enamel matrix derivative (EMD) application following subgingival instrumentation of residual pockets in periodontitis patients on inflammatory host response, microbiological composition, and clinical outcome.

**Methods:**

In this double-blinded randomized controlled trial, a total of 22 patients with generalized periodontitis stage III or IV presenting with ≥ 6 mm probing pocket depth (PPD) at re-evaluation after initial periodontal therapy were included. Participants were randomly allocated at a 1:1 ratio to subgingival instrumentation with (EMD +) or without (EMD-) non-surgical EMD application into the pocket. PPD, clinical attachment level (CAL), bleeding on probing (BoP), plaque index (PI), as well as a panel of pro-inflammatory cytokines and periodontal pathogen count in the gingival crevicular fluid (GCF) of the respective sites were evaluated at baseline (T0) and six months afterwards (T1).

**Results:**

Both treatment groups showed a significant PPD reduction (EMD + 1.33 ± 1.15 mm, *p* < 0.001; EMD- 1.32 ± 1.01 mm, *p* < 0.001) as well as CAL gain (EMD + 1.13 ± 1.58 mm, *p* < 0.001; EMD- 0.47 ± 1.06 mm, *p* = 0.005) from T0 to T1. While no intergroup differences for PPD reduction were observed, CAL gain was higher in EMD + sites compared to EMD- (*p* = 0.009). No essential effects on cytokine expression as well as bacterial count were detected.

**Conclusions:**

Application of EMD as an adjunct to subgingival instrumentation of residual pockets yielded benefits regarding CAL gain; however, effects on PPD reduction, inflammatory cytokines, and bacterial count were negligible.

**Trial registration:**

ClinicalTrials.gov (NCT04449393), registration date 26/06/2020.

**Clinical relevance:**

Based on the obtained results, additional non-surgical EMD application compared to subgingival instrumentation alone showed no clinically relevant effects on treatment outcome and underlying biological mechanisms.

**Supplementary Information:**

The online version contains supplementary material available at 10.1007/s00784-023-05254-1.

## Introduction

Periodontitis is a biofilm-driven inflammatory disease characterized by progressive destruction of the periodontal structures, thereby leading to tooth loss if left untreated [1]. Nonsurgical periodontal therapy (NSPT) aims to eliminate the subgingival bacterial load and to improve clinical parameters such as probing pocket depths (PPD), bleeding on probing (BoP), and clinical attachment levels (CAL) [2, 3]. However, residual pockets with persisting inflammation might still remain at re-evaluation after initial therapy, thereby possibly influencing further disease progression and jeopardizing tooth survival [4]. One treatment approach for residual pockets is to perform resective or regenerative periodontal surgery to achieve resolution of inflammation and, if possible, to restore physiologic anatomy. In this context, it has been shown that periodontal surgery is associated with high dental anxiety scores in patients [5], emphasizing the need for effective non-surgical alternatives. Thus, therapies have been developed to selectively target diseased sites, such as local delivery of antibiotics or antiseptics, the use of laser irradiation, as well as other strategies [6, 7].

For more than 25 years, enamel matrix derivatives (EMD) have been in use as an adjunctive agent during surgical therapy to promote periodontal soft and hard tissue regeneration and have shown beneficial effects such as PPD reduction and CAL gain [8–11]. Studies on nonsurgical (flapless) EMD application as an adjunct to NSPT have so far shown inconsistencies regarding the clinical efficacy, either reporting benefits [12–16] or no additional value [17–21]. Although two recent meta-analyses revealed no significant improvements on PPD and CAL after 3 to 12 months when EMD was used as adjunctive therapy to NSPT, the included studies were of high heterogeneity and authors suggested performing additional RCTs for further assessment [22, 23].

While clinical parameters can be used to assess periodontal disease progression, the underlying inflammatory host immune response is one key aspect promoting tissue destruction [24]. With regard to anti-inflammatory effects, EMD have been demonstrated to reduce interleukin (IL)-1β and receptor activator of NF-κB ligand (RANKL) expression, but also to increase prostaglandin E2 and osteoprotegerin (OPG) expression in vitro [25]. However, clinical data on inflammatory response following non-surgical EMD application in periodontal therapy is scarce and inconsistent**.** Giannopoulou et al. did not observe statistically significant effects on IL-1β, and myeloid-related protein (MRP) 8/14 levels in GCF when EMD was used as an adjunct to subgingival instrumentation [17]. In contrast, Jentsch et al. suggested that additional EMD application following subgingival instrumentation of residual pockets yielded a significant reduction of IL-1β expression in GCF within 12 months [26].

In addition to an overactivated inflammatory response, periodontitis is characterized by a dysbiotic overgrowing subgingival biofilm with increased prevalence of potential periodontal pathogens, dysregulated immune response, and tissue destruction [27]. Spahr et al. have shown in vitro that EMD combined with propylene glycol alginate exert an inhibitory effect on the growth of *Aggregatibacter actinomycetemcomitans*, *Porphyromonas gingivalis*, and *Prevotella intermedia* [28]. In line with these findings, Wyganowska-Świątkowska et al. demonstrated an inhibition of *Porphyromonas gingivalis* and *Prevotella* development when EMD was applied as an adjunct to NSPT in patients undergoing initial periodontal therapy [20].

To the best of our knowledge, the impact of EMD application in the nonsurgical retreatment of remaining periodontal pockets on inflammatory response and subgingival biofilm has so far not been investigated in depth. Thus, the aim of the present study was to assess EMD effects on a broad range of host inflammation biomarkers and bacterial count of periodontitis associated bacteria in addition to clinical outcome in periodontitis patients presenting with residual pockets at re-evaluation. The null-hypothesis of the present study was that the use of EMD in addition to subgingival instrumentation would have no beneficial effects on the clinical outcome, inflammation, and bacterial count when compared to subgingival instrumentation alone.

## Materials and methods

### Patient selection and study design

For this prospective, 1:1 randomized, double-blinded, controlled clinical trial, patients were consecutively recruited from September 2020 to May 2022 at the Division of Conservative Dentistry and Periodontology, Medical University of Vienna, Austria. The ethics committee (Ethics Commission of the Medical University of Vienna) provided approval for this study (1248/2020), and the study was registered at clinicaltrials.gov (https://clinicaltrials.gov/ct2/show/NCT04449393). The study was conducted in accordance with the Declaration of Helsinki of 1975, as revised in 2013, and in agreement with CONSORT guidelines [29] and the “Good Scientific Practice” guidelines of the Medical University of Vienna. Informed consent was obtained from each patient prior to commencement of the study. Screening for eligibility was performed in patients with already finished initial periodontal therapy and after re-evaluation, who met the following inclusion criteria: (1) At least one site with a probing depth of ≥ 6 mm about 8 weeks after step II of periodontal therapy at re-evaluation; (2) periodontitis stage III or IV [30]; (3) age between 25 and 75; and (4) no antibiotic therapy during the last 3 months. The exclusion criteria were pregnancy, lactation, and systemic diseases which potentially could influence outcome of the therapy (e.g., diabetes mellitus with HbA1c > 7.5%, immunosuppression, malignant diseases, and rheumatoid arthritis).

**Fig. 1:**
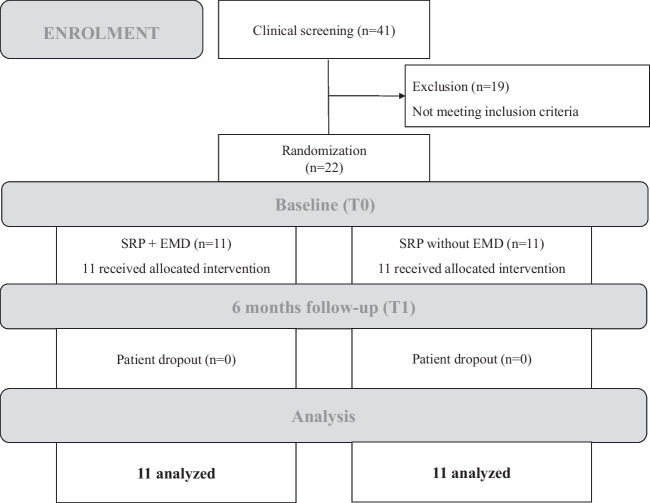
CONSORT flow chart of the study design. Evaluation of clinical parameters (PD, CAL, PI, BOP), oral hygiene indices (API, PBI), cytokine analysis, and microbial assessments were performed at baseline and at 6-month follow-up

Of 41 patients that were assessed for eligibility, 19 did not meet inclusion criteria. A total of 22 systemically healthy patients were enrolled as per protocol and randomly assigned to the test group, who received EMD (EMD + ; *n* = 11) and to the control group, who did not receive EMD (EMD-; *n* = 11). The 1:1 randomization was performed using online available tool (www.randomizer.org). Patients were blinded to treatment. In both groups, evaluation of clinical parameters (PD, CAL, PI, BOP), oral hygiene indices (API, PBI), cytokine analysis, and microbial assessments were performed at baseline (T0) and at 6 months follow-up (T1). The general study flow diagram is presented in Fig. 1.

### Clinical examination and treatment

Periodontal treatment consisted of whole-mouth supragingival debridement applying sonic debridement (Sonicflex® T KaVo, Biberach, Germany) as well as erythritol powder air polishing (AIRFLOW® Prophylaxis Master, E.M.S. Electro Medical Systems S.A, Nyon, Switzerland). Subgingival instrumentation of sites with PPD ≥ 6 mm was conducted with Gracey curettes (*Hu-Friedy,* Chicago, IL, USA) in combination with sonic debridement (Sonicflex® T KaVo, Biberach, Germany).

At the completion of subgingival instrumentation for participants in test group (EMD +), a 24% EDTA solution (Prefgel®; Institute Straumann AG, Basel, Switzerland) was inserted into the gingival sulcus using prefilled sterile syringes. The sites were then rinsed with saline solution. After irrigation, sterile gauze was used to prevent saliva contamination of the selected sites, followed by gentle air-drying. Orthodontic floss (Superfloss®; Oral B, Ireland) was placed and left in the site for 1 min. Irrigation and floss application were repeated until complete bleeding control. Afterwards, EMD (Emdogain® FL; Institute Straumann AG, Basel, Switzerland) was applied on each site, until overflow of the gel from the marginal gingiva was achieved. In control group patients (EMD-), saline solution was applied after subgingival instrumentation. Clinical procedures were performed without local anesthesia.

All patients were instructed to apply chlorhexidine gel (Chlorhexamed 1% Gel, GlaxoSmithKline, Brentfort, UK) at the treated sites for 2 weeks and to refrain from brushing and flossing at the respective areas. At weeks 3 and 4, patients were told to use a soft toothbrush for the treatment sites. Afterwards, oral hygiene was performed as usual. Treatment procedures were performed by two certified examiners with a specialization in periodontology (CW, GD). Re-assessment was performed 6 months post-treatment by a third examiner who was blinded to the treatment method (DT).

All three examiners involved in study procedures were calibrated for inter-examiner reliability assessing PPD in 42 randomly chosen sites. Measurements were performed with a calibrated standard probe. Intraclass correlation coefficient (ICC) was 0.979 (95% CI 0.965–0.988; *p* < 0.001) for PPD measurements.

### PICF collection

PICF samples were collected at baseline (T0) and 6 months after treatment (T1). The respective sites were gently air-dried and isolated with sterile gauze to prevent contamination with saliva. A sterilized paper collection strip (PerioPaper strips, Oraflow Inc, Plainview, NY, USA) was inserted into the gingival sulcus of the experimental site until slight resistance for 30 s. Samples were disposed in case contamination with blood was observed. A calibrated electronic volume quantification device (Periotron 8000, Oralflow Inc, Plainview, NY, USA) was used for volumetric assessment. Paper strips from each site were put in Eppendorf tubes and subsequently stored at − 80 °C until further analysis. Protein extraction was performed after sample thawing according to an elution procedure specified previously [31]. A 20 μl of extraction buffer (24.5 mL PBS (Phosphate Buffered Saline, pH 7.4), 125 ml phenylmethylsulfonylfluoride (PMSF; Sigma Chemical, St. Louis, MO), 200 mM in Methanol, 1 mg/ml in water, and 83.5 ml of 30% Human Serum Albumin (Sigma Chemical, St. Louis, MO) were pipetted onto the cellulose component of the collection strip. Centrifugation of the strips placed in Eppendorf tubes was conducted at 2000 rpm at 4 °C for 5 min. This procedure was repeated for 4 times to obtain a total volume of 100 μl for each tube. Afterwards, the entire product was then stored on dry ice for evaluation of biomarker concentration.

### Cytokine analysis

A multiplex ELISA kit (Quantibody Human Periodontal Disease Array 1 Kit, RayBiotech, Norcross, GA, USA) was used to assess the expression of C-reactive protein (CRP), interferon (IFN)-γ, tumor necrosis factor (TNF)-α, transforming growth factor (TGF)-β, interleukin (IL)-1α, IL-1β, IL-2, IL-4, IL-6, IL-8, IL-10, IL-12A, IL-17A, macrophage inflammatory protein (MIP)-1α*,* matrix metalloproteinase (MMP*)-*9, MMP-13, osteopontin, osteoactivin, and osteoprotegerin. Levels of receptor activator of NF-κB ligand (RANKL) were assessed by ELISA (RayBiotech, Norcross, GA, USA). Concentrations were determined by generating a standard curve for calibration.

### Microbiologic examination

Prior to subgingival instrumentation, subgingival plaque samples were collected using sterile paper points that were inserted into the respective sites for 10 s. The paper points were pooled in Eppendorf tubes and transported to the microbiology laboratory for further processing. Eleven putative periodontal pathogens (*A. actinomycetemcomitans*, *P. gingivalis*, *T. forsythia*, *T. denticola*, *P. intermedia*, *P. micros*, *F. nucleatum*, *C. rectus*, *E. nodatum*, *E. corrodens*, *Capnocytophaga species*) were assessed using commercially available polymerase chain reaction (PCR) DNA probe test kit (micro-IDent Plus, Hain Lifescience, Nehren, Germany) following manufacturer’s instructions.

### Primary and secondary outcomes

PPD was defined as primary outcome variable. Secondary outcomes of interest were CAL, BoP, API, PBI, and number of pocket sites with closed pockets (PPD ≤ 4 mm without BoP) and residual pockets (PPD ≥ 5 mm regardless of BoP) as well as parameters from the cytokine and microbiologic analysis.

### Statistical analysis

Assuming a fairly normal distribution of the sample and a clinically significant difference in PPD of 0.7 mm with a standard deviation of 0.5 mm between EMD + and EMD-, the calculation showed that at least 10 patients per group would achieve a power of 85% at a significance level of α = 0.05. To compensate for the possible loss of patients, a sample size of 22 patients with 11 patients in each group were recruited.

Descriptive statistic was used to present patient characteristics. Data distribution was assessed by visual inspection of histograms and the Kolmogorov–Smirnov test, and the Levene’s test was used for the equality of variances. All continuous data were presented as means with standard deviation and categorical variables as proportions and frequency counts.

Independent *t*-tests or the non-parametric Mann–Whitney *U* tests were used to compare continuous variables (e.g., cytokines) between sites treated with (EMD +) and without EMD (EMD-). Non-parametric Wilcoxon signed-rank tests were used to compare quantitative data (e.g., levels of single parameter in PICF samples) between baseline (T0) and the 6-month time point (T1). Categorical data were assessed using Chi-square or Fisher’s exact tests between EMD + and EMD-. McNemar was used to compare categorical data between T0 and T1. Analysis of covariance (ANCOVA) models, adjusted for T0 of T1 PPD and CAL means, were developed. All data were analyzed using SPSS software (PAWS Statistics 26; SPSS Inc., Chicago, IL). Statistical significance was set at the conventional *P* value of < 0.05 (two-sided).

## Results

Demographics of the 22 participants (11 treated with EMD and 11 without EMD) including age (EMD + 47.4 ± 10.9 years, EMD- 51.8 ± 10.2 years; *p* = 0.349), gender (EMD + 8 female/3 male, EMD- 5 female/6 male; *p* = 0.193), and smoking status (EMD + 45.5% smoker, EMD- 36.4% smoker; *p* = 0.665) were similar between the groups. A total of 89 sites (45 with EMD and 44 without EMD) were evaluated. The majority of patients (98.9%) had more than one site involved. All study participants were diagnosed with periodontitis Stage III. Presence of vertical bone defects was comparable between EMD + (55.6%) and EMD- (43.2%; *p* = 0.243). No adverse effects or side effects were observed during the observation period and at regular follow-up appointments. Demographics and information regarding site distribution are shown in Table 1.

**Table 1:** Patient characteristics and site distribution

	EMD + (*n* = 11)	EMD- (*n* = 11)	*P*-value (between groups)
Patient characteristics			
Age (mean ± SD)	47.4 ± 10.9	51.8 ± 10.2	0.349
Gender (female;*n*(%))	8 (72.7)	5 (45.5)	0.193
Smoking status (smoking; *n*(%))	5 (45.5)	4 (36.4)	0.665
Site distribution			
Total number of sites (n)	45	44	
Patients with one site involved (n)	0	1	
Patients with two site involved (n)	2	0	
Patients with three site involved (n)	1	2	
Patients with four site involved (n)	5	6	
Patients with five site involved (n)	2	1	
Patients with eight site involved (n)	1	1	

### Clinical outcomes

Clinical parameters of included sites (*n* = 89) compared between EMD + and EMD- are presented in Table 2. PPD and CAL significantly improved in both groups from T0 to T1. The mean PPD reduction was 1.33 ± 1.15 mm for EMD + sites and 1.32 ± 1.01 mm for EMD- sites (both *p* < 0.001). The mean CAL gain was 1.13 ± 1.58 mm for experimental sites (*p* < 0.001) and 0.47 ± 1.06 mm for control sites (*p* = 0.005), with significant better outcome in the EMD + group compared to EMD- (*p* = 0.009).

**Table 2:** Comparison of clinical parameters between sites treated with and without EMD

		EMD + ( 45 sites)	EMD-( 44 sites)	*P*-value (between groups)
PPD (mm; mean ± SD)	T0	6.31 ± 0.73	6.49 ± 0.79	0.196
	T1	4.98 ± 1.18	5.17 ± 0.86	0.255
	*p*-value (within group)	< 0.001	< 0.001	
	Difference (T1-T0)	-1.33 ± 1.15	-1.32 ± 1.01	0.880
CAL (mm; mean ± SD)	T0	7.20 ± 1.38	7.39 ± 0.99	0.281
	T1	6.07 ± 1.53	6.92 ± 1.29	0.003
	*p*-value (within group)	< 0.001	0.005	
	Difference (T1-T0)	-1.13 ± 1.58	-0.47 ± 1.06	0.009
BOP (% of all sites)	T0	64.4	47.7	0.112
	T1	28.9	15.9	0.142
	*p*-value (within group)	< 0.001	0.007	
Plaque index (% of all sites)	T0	33.3	25.0	0.387
	T1	26.7	31.8	0.593
	*p*-value (within group)	0.508	0.581	
Closed pockets	% of all sites with PPD ≤ 4 mm no BoP (T1)	20.5	20.0	0.957
Residual pockets	% of all sites with PPD ≥ 5 mm ± BoP (T1)	75.6	79.5	0.652

Site-specific BoP was reduced significantly during the observation period in EMD- patients (from 47.7 to 15.9%, *p* = 0.007), as well as in the EMD + group (from 64.4 to 28.9%, *p* < 0.001). Site-specific PI as well as percentages of closed or residual pockets did not reveal significant changes at T1 compared to T0 and showed no intergroup differences. Sixteen sites reached pocket closure (PPD ≤ 4 mm without BoP) after treatment; one 5 mm site (EMD + group), eleven 6 mm sites (EMD- 7, EMD + 4), three 7 mm sites (EMD- 1, EMD + 2), and one 8 mm site (EMD- group).

### Oral hygiene indices

Oral hygiene indices approximal plaque index (API) and papillary bleeding index (PBI) for the test and control group are displayed in Table 3. At the 6 months follow-up, API was significantly higher in test group patients compared to control (44.1 ± 16.9% vs. 31.7 ± 4.8%, respectively, *p* = 0.045). No intragroup differences were observed for API and PBI in the course of the study.

**Table 3:** Comparison of oral hygiene indices between patients treated with and without EMD

		EMD + (*n* = 11)	EMD-(*n* = 11)	*P*-value (between groups)
API	Baseline (T0)	40.2 ± 16.0	37.4 ± 15.9	0.511
	6-months follow-up (T1)	44.1 ± 16.9	31.7 ± 4.8	0.045
	*p*-value (within group)	0.401	0.441	
	Difference (T1-T0)	3.9 ± 17.8	-5.7 ± 16.0	0.176
PBI	Baseline (T0)	7.0 ± 9.3	9.6 ± 10.2	0.523
	6-months follow-up (T1)	4.9 ± 7.2	3.4 ± 4.8	0.774
	*p*-value (within group)	0.400	0.066	
	Difference (T1-T0)	-2.1 ± 9.1	-6.3 ± 9.9	0.301

### Effects on inflammation biomarkers in GCF

**Fig. 2:**
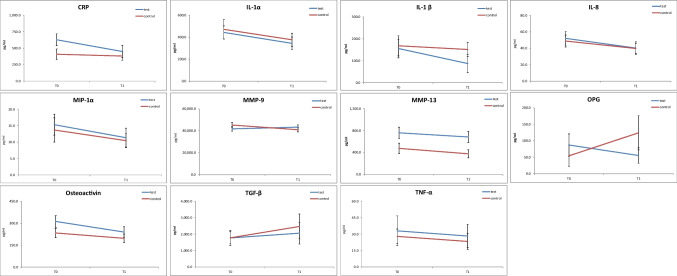
Effects on pro-inflammatory cytokine expression

Levels of pro-inflammatory cytokines in EMD + vs. EMD- group are displayed in Fig. 2. CRP, IL-1α, IL-8, MIP-1α, MMP-13, osteoactivin, and TNF-α showed a tendency for decrease at T1 compared to T0 which was not statistically significant. Levels of MMP-13 were statistically higher in EMD + group sites compared to EMD- (*p* = 0.03). Comparing baseline to 6 months follow-up, MMP-9 levels slightly increased in test group whereas declined in control, both not meeting the criteria for significance. TGF-β levels raised insignificantly during the observation period in both groups. OPG levels tended to increase in control and decrease in test group without statistical significance. No intergroup differences in cytokine expression were observed. Interferon gamma (IFN*-*γ), IL-2, IL-4, IL-6, IL-10, IL-12, IL-17, OPN, RANK, and RANKL were below the detection limit in the majority of samples and therefore were excluded from analysis.

### Effects on bacterial count

**Fig. 3:**
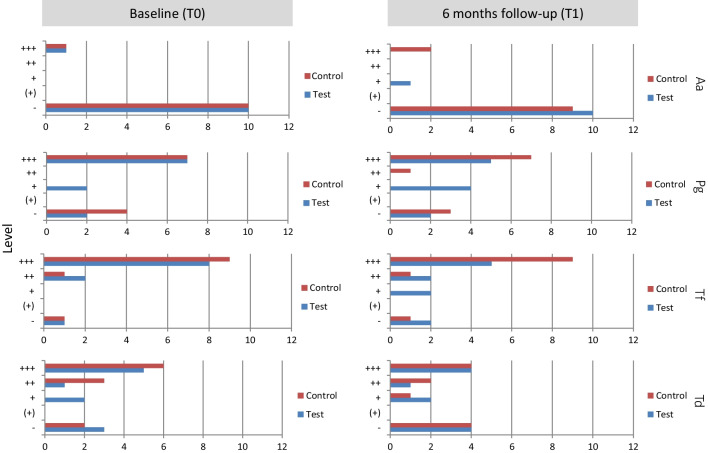
Effects on bacterial count

Impact on periodontal pathogen count is shown in Fig. 3. A tendency for bacterial count reduction in GCF was observed for *A. actinomycetemcomitans*, *P. gingivalis*, *T. forsythia*, and *T. denticola* at 6 months follow-up compared to baseline. Statistical significance was not detected.

## Discussion

Statistically significant changes in PPD were observed in both EMD + and EMD- groups from baseline to 6 months. However, the present study results suggest that the adjunctive, flapless use of EMD in the treatment of residual pockets does not additionally improve PPD reduction obtained with subgingival instrumentation alone. Furthermore, BoP was significantly reduced in EMD + (from 64.4% to 28.9%, *p* < 0.001) and EMD- (from 47.7% to 15.9%, *p* = 0.007), while no intergroup differences were observed. These findings are in line with a number of previous investigations evaluating nonsurgical EMD use during initial periodontal therapy [18, 19, 32, 33] as well as in periodontal maintenance patients [21].

With regard to CAL gain, the EMD + group (1.13 ± 1.58 mm) was significantly better compared to subgingival instrumentation alone (0.47 ± 1.06 mm), which was not observed in the abovementioned studies. This might be due to a different clinical scenario, as patients in the present trial had already undergone steps 1 and 2 of periodontal therapy (supra- and subgingival biofilm removal) recently, thus reflecting a later stage of periodontal treatment. On the other hand, a study by Jasa et al. included patients with a history of regular periodontal maintenance, again representing a different patient collective. The present study results suggest potential benefits of flapless EMD application on CAL gain rather than PPD reduction when applied for the treatment of remaining diseased sites at re-evaluation after initial periodontal therapy. This indicates that the effects of EMD seem to be more related to maintaining the levels of the gingival margin which could be due to the reduced level of inflammation and therefore less soft tissue shrinkage during the healing process.

Interestingly, API was higher in EMD + compared to EMD- patients at the 6 months follow-up (44.1 ± 16.9% vs. 31.7 ± 4.8%, respectively; *p* = 0.045), while both groups had comparable values at baseline. However, no significant alterations of both API and PBI were detected within both groups during the observation time, indicating that both treatment protocols had no substantive impact on oral hygiene parameters.

According to our results, no essential reduction of bacterial count in residual pockets was observed following subgingival instrumentation combined with flapless EMD application compared to subgingival instrumentation alone. This is in contrast to Wyganowska-Świątkowska who found that the use of EMD as an adjunct to subgingival instrumentation during initial periodontal therapy inhibited the growth of Gram-negative bacteria including *Porphyromonas gingivalis* and *Prevotella intermedia* [20]. However, the respective investigation again represents an earlier treatment stage than the current study setting. Although mechanical debridement of the root surface has shown high efficacy in biofilm elimination and reduction of subgingival microflora during initial periodontal therapy [34], this effect might be less present in residual pockets that have recently been treated. Also, in the current study setting, 6 months between treatment and re-evaluation might be too long to detect significant differences on bacteria count. In this context, it might be worth evaluating possible effects of EMD application on bacterial reduction at earlier time points. Moreover, another reason for our results might be that participants had to apply chlorhexidine gel at the treatment sites, which could have reduced bacterial count in both groups.

The present findings also indicate no substantial influence of non-surgical EMD application on inflammatory markers in GCF. These results confirm findings by Giannopoulou et al. who assessed IL-1 expression at protein level in GCF 2 months after nonsurgical periodontal treatment with and without adjunctive EMD application [17]. Although several tendencies for a reduction in pro-inflammatory cytokines were observed in the present study, they did not meet the level of significance. While EMD has been shown downregulate the expression of genes associated with early inflammation in PDL cells in vitro [35], this potential anti-inflammatory effect did not translate into clinical conditions under the current treatment setting.

The present clinical investigation had some limitations. The limited sample size might have prevented further findings on clinical parameters, as well as on cytokine or bacterial levels. Focus on retreatment of periodontal sites that have already undergone initial periodontal therapy without sufficient site-specific success might have resulted in less improvement of clinical parameters when compared to first phase interventions. The inclusion of groups with application of PGA and/or 24% EDTA solution only might also provide further information, as PGA has also antimicrobial properties. Moreover, measurements of the assessed inflammatory and bacterial parameters at different time points might also elucidate effects of EMD application. Further research is needed to determine whether clinical benefit of nonsurgical EMD application might be influenced by site-specific conditions or treatment settings. Also, direct comparison of treatment outcome of surgical and nonsurgical application of EMD should be encouraged.

## Conclusion

The present study indicates an improvement of CAL gain following non-surgical EMD application compared to subgingival instrumentation alone for the treatment of residual periodontal pockets at re-evaluation. GCF analysis revealed that adjunctive nonsurgical use of EMD does not induce essential effects on pro-inflammatory cytokine expression as well as bacterial count reduction.

### Supplementary Information

Below is the link to the electronic supplementary material.

Supplementary file1 (PDF 231 KB)

## Data Availability

The data will be available when requested to the corresponding author.
