# Supplementary material for: Effects of enamel matrix derivative in nonsurgical periodontal therapy on pro-inflammatory profiles, microbial environment and clinical outcome: a randomized clinical trial
Source: Clin Oral Investig. 2023 Oct 16;27(11):6493–502. doi: 10.1007/s00784-023-05254-1 (PMC10630232; doi:10.1007/s00784-023-05254-1)
Supplement: Supplementary file 1 — Supplementary file1 (PDF 231 KB) [file 784_2023_5254_MOESM1_ESM.pdf]

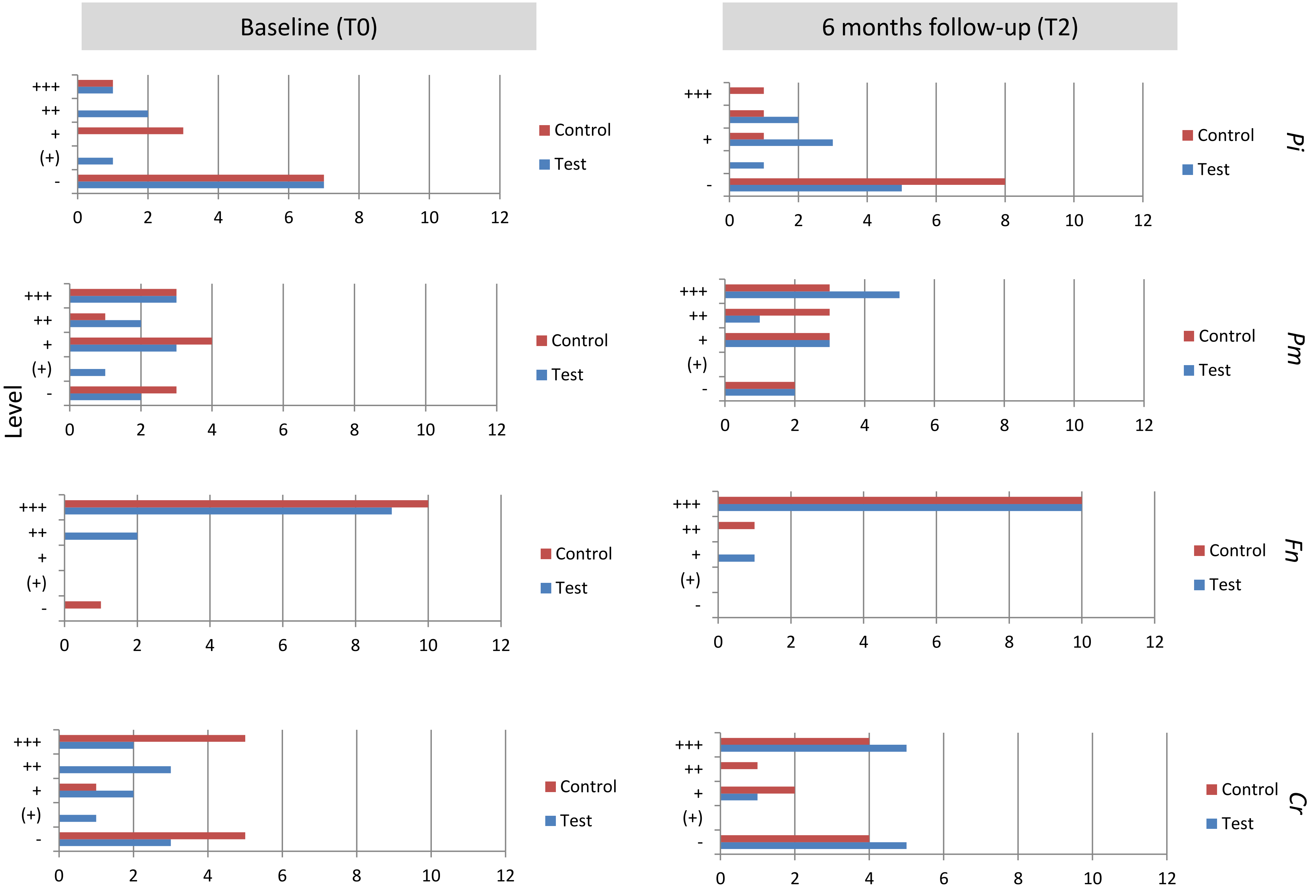

Baseline (T0)
6 months follow-up (T2)
Level
+++
++
+
(+)
-
0
2
4
6
8
10
12
Control
Test
Pi
Pm
Fn
Cr

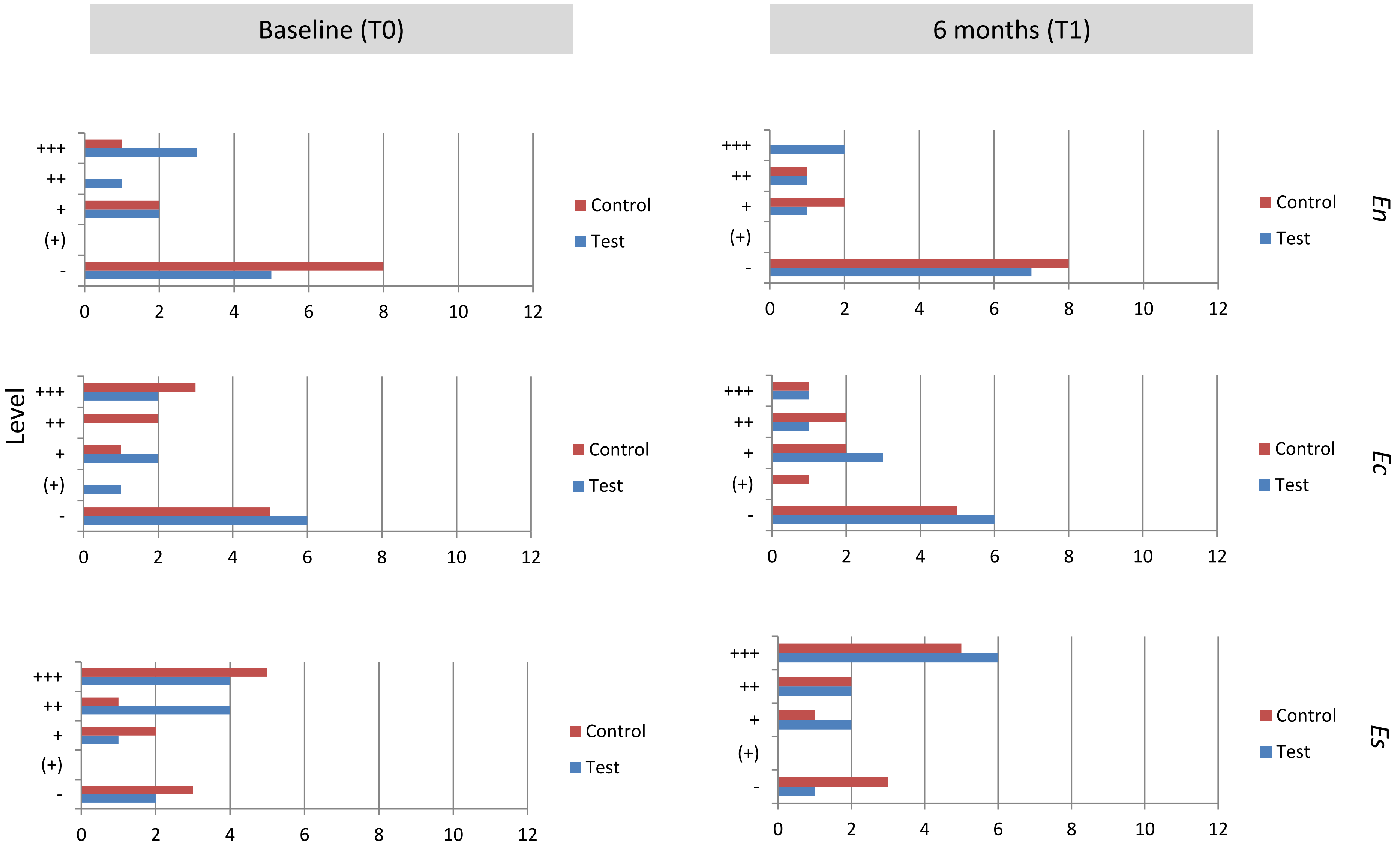
Baseline (T0)
6 months (T1)
Level
+++
++
+
(+)
-
0
2
4
6
8
10
12
Control
Test
En
Ec
Es
